# Supplementary material for: vPro-MS enables identification of human-pathogenic viruses from patient samples by untargeted proteomics
Source: Nat Commun. 2025 Jul 31;16:7041. doi: 10.1038/s41467-025-62469-4 (PMC12314097; doi:10.1038/s41467-025-62469-4)
Supplement: Supplementary file 1 — Supplementary Information [file 41467_2025_62469_MOESM1_ESM.pdf]

# Suspension Trap (STrap) Sample Preparation for Virus Identification from Swab Samples

## 1. Materials

### Solutions and Reagents

- Phosphate Buffered Saline (store at 4°C)
- SDS stock solution: 20% SDS
- Reduction/alkylation buffer (10x): 100 mM Tris(2-carboxyethyl)phosphine (TCEP, 29 mg/mL), 400 mM 2-Chloroacetamide (CAA, 37 mg/mL) in 200 mM Tris/TFA (pH = 8.5)
- Binding/wash buffer: 100 mM TEAB (final) in 90% methanol (1 year at 4°C)
- Acidifier: 27.5% phosphoric acid (sealed 1 year at RT)
- Enzyme Solution: Trypsin Gold 1 µg/µL in 50 mM TEAB (store at -80°C)
- Digestion buffer: 50 mM TEAB
- Elution buffer: 50% acetonitrile
- Resuspension buffer: 0.1% formic acid
- Water

### Equipment

- Bench-top centrifuge
- ThermoMixer
- UV-Spectrophotometer (e.g. Nanodrop)
- Eppendorf Protein LoBind tubes (1.5 mL)
- SpeedVac
- S-Trap™ micro (Protifi)

## 2. Swap Preparation

1. Add 200 µL **PBS** to a 1.5 mL tube
2. Cut off the **swab** right after the foam and add it to the tube.
3. Vortex sample thoroughly
4. Incubate sample for 1 min
5. Remove **swab** from the **PBS**
6. Centrifuge at 20,000 g for 10 min
7. Save supernatant in a new tube
8. Store sample at -20°C until further use

## 3. Protein Digestion using S-Trap™ micros

*After each centrifugation step make sure that all added solution has gone through the S-Trap*

*Preheat digestion buffer without trypsin to 47°C*

1. Transfer 20 µL sample to a new tube
2. Add 2 µL **20% SDS**
3. *Optional: Add 2 µL* ***reduction/alkylation buffer***
4. Incubate samples at **95°C for 5 min**

*optional: Clarify lysate by centrifugation at 16,000 x g for 5 min.*

5. Add 2.5 µL **acidifier** (pH must ≤ 1) and vortex
6. Add 165 µL of **binding buffer** and vortex
7. Place S-Trap in a tube and apply sample to column
8. Centrifuge at 4,000 g for 30 s.
9. Add 200 µL **wash buffer** and centrifuge at 4,000 g for 30 s
10. Repeat wash 2 times and discard flow through as necessary

*Rotate the S-Trap units 180 degrees between the centrifugations.*

11. Place S-Trap in a **new tube** for digestion
12. Add 20 µL of **digestion buffer** containing 2 µg **trypsin**
13. Centrifuge at 100 g for 2 s to remove air bubbles
14. Cap the S-Trap loosely to limit evaporative loss

*Do not make an airtight seal with the cap, as this will force the solution out of the S-Trap.*

15. Incubate for **1 h at 47 °C** without shaking
16. Add 40 µl **water** and centrifuge at 4,000 g for 30 s
17. Add 40 µl **elution buffer** and centrifuge at 4,000 g for 30 s
18. Dry down sample in a SpeedVac
19. Resuspend peptides in 20 µL **resuspension buffer**
20. Store peptides at – 20°C.
