# Supplementary material for: vPro-MS enables identification of human-pathogenic viruses from patient samples by untargeted proteomics
Source: Nat Commun. 2025 Jul 31;16:7041. doi: 10.1038/s41467-025-62469-4 (PMC12314097; doi:10.1038/s41467-025-62469-4)
Supplement: Supplementary file 2 — Description Of Additional Supplementary File [file 41467_2025_62469_MOESM2_ESM.pdf]

Description of Additional supplementary files

Supplementary Data 1:
Lab protocol for STrap based sample preparation of swab samples

Supplementary Data 2:
List of viruses in the human virome peptide spectral library

Supplementary Data 3:
vPro Library Taxonomic Summary

Supplementary Data 4:
Top ranked virus proteomes of sample T17 prior to confidence filtering

Supplementary Data 5:
Comparison of proteomics and mNGS for the detection of SARS-CoV-2 in swab samples using untargeted workflows

Supplementary Data 6:
Sample List Specificity Panel

Supplementary Data 7:
Sample List Sensitivitiy Panel - SARS-CoV-2
